# *HSP70* and *TNF* Loci Polymorphism Associated with the Posner-Schlossman Syndrome in a Southern Chinese Population

**DOI:** 10.1155/2022/5242948

**Published:** 2022-12-09

**Authors:** Wei Bai, Xiaosheng Huang, Xiaoli Shen, Ye Ye, Shiming Peng, Tianhui Zhu, Shaoyi Mei, Jiajie Kuang, Sejie Yu, Xiaochen Ma, Jun Zhao

**Affiliations:** ^1^The Second Clinical Medical College, Jinan University, Shenzhen 518020, China; ^2^Shenzhen Eye Institute, Shenzhen Eye Hospital, Jinan University, Shenzhen 518040, China; ^3^Department of Ophthalmology, Shenzhen People's Hospital (The Second Clinical Medical College), Jinan University, The First Affiliated Hospital, Southern University of Science and Technology, Shenzhen 518020, China

## Abstract

Previous studies have shown that *HLA* gene polymorphisms are associated with the pathogenesis of the Posner-Schlossman syndrome (PSS). This study was aimed at evaluating the associations between *HLA-III* gene polymorphisms and PSS in a southern Chinese Han population. A total of 150 PSS patients and 183 healthy controls were included in this study. Twenty-one single nucleotide polymorphisms (SNPs) of *HLA-III* genes (including *HSP70-1*, *HSP70-2*, *HSP70-hom*, *TNF-α*, *TNF-β*, *C2,* and *CFB*) were genotyped using the SNaPshot technique. Our study showed that the frequencies of G allele at rs909253, A allele at rs1041981, and G allele at rs2844484 of *TNF-β* in the patient group were significantly higher than those in healthy controls (Corrected *P* (*P*_*c*_) = 0.040, OR = 1.45; *P*_*c*_ = 0.033, OR = 1.45; *P*_*c*_ = 0.045, OR = 1.58, respectively). The frequency of T allele at rs12190359 of *HSP70-1* was significantly lower in PSS patients than those in healthy controls (*P*_*c*_ = 0.018 and OR = 0.10). The frequencies of the CCT haplotype of *HSP70-1* gene (rs1008438-rs562047-rs12190359) and the ACCCTTT haplotype of *HSP70* gene (rs2227956-rs1043618-rs1008438-rs562047-rs12190359-rs2763979-rs6457452) were significantly lower in PSS patients than those in healthy controls (*P*_*c*_ = 0.024, OR = 0.10; *P*_*c*_ = 0.048, OR = 0.10, respectively). In conclusion, the G allele at rs909253, A allele at rs1041981, and G allele at rs2844484 of *TNF-β* gene might be risk factors for PSS, while the T allele at rs12190359 of *HSP70-1* gene and specific haplotypes of the *HSP70-1* and *HSP70* genes might be protective factors for PSS.

## 1. Introduction

The Posner-Schlossman syndrome (PSS) was first reported by Posner and Schlossman in 1948, which is an eye disease with recurrent unilateral nongranuloma anterior uveitis with elevated intraocular pressure (IOP) [1, 2]. The pathogenesis of PSS is still unclear, mainly including the theory of pathogenic microbial infection, autoimmune and endocrine theory, vascular origin theory, and allergy theory [2–5]. The pathogens associated with PSS have been reported to be cytomegalovirus (CMV), varicella-zoster virus, herpes simplex virus, and helicobacter pylori [6, 7].

Human leukocyte antigen (HLA) genes include a series of genes closely related to human immune function, which have various functions such as immune recognition, immune surveillance, antigen presentation in immune response, and regulation of specific immune response [8]. Previous studies have found that *HLA* gene polymorphisms are associated with the pathogenesis of PSS. Hirose et al. found that HLA-Bw54 antigen and haplotype HLA-Bw54-Cwl frequencies of Japanese PSS patients were significantly increased, suggesting for the first time that immune or immune genetic factors might play a role in the pathogenesis of PSS [9]. In previous studies, we found that the *HLA-I* and *-II* polymorphisms were associated with PSS, such as *HLA-C*^∗^*14 : 02* and *HLA-E*^∗^*01 : 03* alleles, and the *HLA-A*^∗^*11 : 01-C*^∗^*14 : 02*, *HLA-B*^∗^*51 : 01-C*^∗^*14 : 02,* and *HLA-E*^∗^*01 : 03-G*^∗^*01 : 01* haplotypes might be risk factors for PSS pathogenesis, while the *HLA-DPA1*^∗^*02 : 01* and *HLA-DPB1*^∗^*17 : 01* alleles and the *HLA-B*^∗^*13 : 01-C*^∗^*03 : 04* and *HLA-DPB1*^∗^*14 : 01-DPA1*^∗^*02 : 01* haplotypes might be protective factors for PSS [10–12]. To date, the relationship between *HLA-III* genes and the Posner-Schlossman syndrome in southern Chinese Han population remains unknown. *HLA-III* genes are physically located between *HLA-I* and *HLA-II* genes, which are the most concentrated region of gene distribution, including complement (*C*) *2*, *C4*, *CFB,* heat shock proteins (*HSPs*) *-70*, and tumor necrosis factor (*TNF*) *-α* and *-β* [13].

The HSP70 family is composed of HSP70-1 (HSPA1A), HSP70-2 (HSPA1B), and HSP70-hom (HSPA1L). The *HSP70-1* and *HSP70-2* genes encode the same protein product of 641 amino acids, both the expression of HSP70-1 and HSP70-2 was heat-induced. The sequences of the 3′ untranslated regions of the *HSP70-1* and *HSP70-2* genes are completely different, which may have different mRNA transcriptional regulation. *HSP70-hom* gene encodes a more basic protein that is not thermally induced and is highly related to *HSP70-1* [14]. Ayub et al. and Salehi et al. showed that G/C and C/C genotypes at rs1043618 of *HSP70-hom* and G/G genotype at rs1061581 of *HSP70-2* were associated with increased risk of glaucoma in the Pakistani and the Iranian population respectively [15, 16]. TNF-*α* is a potent proinflammatory cytokine playing an important role in inflammatory and immune responses [17]. Previous studies have found that TNF-*α* played an important role in the occurrence and development of uveitis by affecting the transcription regulation and protein expression levels of TNF-*α* [18]. Xin et al. showed that the G/A genotype at rs1800629 of *TNF-α* increases the risk of glaucoma in Asian patients [19]. Wang et al. found that A allele at rs645836 of *TNF-α* might be a protective factor of primary open-angle glaucoma [20]. Persistent production of TNF-*α* occurs in many autoimmune inflammatory diseases, including uveitis, and this is associated with significant tissue damage. Although uveitis represents a phenotypically heterogeneous group of intraocular inflammatory conditions, they have in common raised levels of TNF-*α* in both serum and aqueous humor [21]. TNF-*β* is a kind of cytokine produced in parts of autoimmune diseases and tumors after the stimulation of mitogen and lymphocyte antigen. Since having similar structure to the TNF-*α*, TNF-*β* belongs to the TNF family, and it can affect cell apoptosis and regulate inflammatory immunity [22]. Our previous studies found that the serum concentrations of C3, C4, C1q, FCN2, C3a, and sC5b-9 in the PSS patients were significantly higher than those in the normal control group, suggesting that the complement replacement pathway might be abnormally activated in PSS patients at onset [23].

In this study, 21 loci of class *HLA-III* genes were genotyped and analyzed in a group of PSS patients and controls from the southern Chinese Han population to evaluate the association of *HLA-III* with PSS.

## 2. Methods

### 2.1. Subjects

A total of 150 unrelated patients with PSS from the southern Chinese Han population were recruited from the Shenzhen Eye Hospital in China from 2018 to 2021 in this study. Patients were diagnosed with PSS according to the following classical criteria and amendments [2, 24]: (1) there were unilateral recurrent episodes of IOP elevation over 21 mmHg, and the IOP may reach 40 mmHg or more; (2) a few white keratic precipitates (KPs) accumulating in the lower half of the cornea and the mild inflammatory reaction in the anterior chamber; (3) open chamber angle and no iris posterior adhesion; (4) no significant decrease or slight decrease in visual acuity, no visual field loss, and optic nerve damage in patients with shorter course of disease; (5) no history of other eye diseases except for refractive error. One hundred and eighty-three unrelated subjects were recruited at the Shenzhen Blood Center from healthy volunteer blood donors without eye disease. Immune system abnormalities and a history of tumor were excluded in all subjects. Patients and controls were all southern Han Chinese and matched for age, sex, and ethnicity.

### 2.2. Single Nucleotide Polymorphisms Selected, Sample Collection, DNA Extraction, Polymerase Chain Reaction Amplification and Genotyping

Previous studies have found that the *HLA-I* and *-II* polymorphisms are associated with PSS. A number of studies have also shown that polymorphisms of many *HLA-III* genes are related to the development of uveitis and glaucoma [15–23, 25, 26]. Considering that PSS shares some common features of glaucoma and uveitis, we wondered whether PSS was also associated with *HLA-III* genes, so we selected 21 SNPs in functional regions of *HLA-III* genes for analysis. Twenty-one SNPs in *HSP70-1*, *HSP70-2*, *HSP70-hom*, *TNF-α*, *TNF-β*, *CFB,* and *C2* in the *HLA-III* loci were genotyped in PSS patients and healthy controls (Supplementary Table 1). Peripheral blood samples were collected in ethylenediaminetetraacetic acid (EDTA) anticoagulant tubes from all study participants. Blood samples were stored at -80°C for further analysis.

Genomic DNA was extracted from blood samples using the TIANamp Blood DNA Kit (Tiangen Biotechnology, Beijing, China). The total volume of the polymerase chain reaction (PCR) amplification was 20 *μ*L, including 1 *μ*L genomic DNA, 1 *μ*L for each PCR primer, 1× HotStarTaq buffer, 3.0 mM Mg^2+^, 0.3 mM dNTP, and 1 U HotStarTaq polymerase (Qiagen, Hilden, Germany). PCR amplification systems B and C included 1 *μ*L genomic DNA, 1 *μ*L for each PCR primer, 1× GC-I buffer, 3.0 mM Mg^2+^, 0.3 mM dNTP, and 1 U HotStarTaq polymerase (Qiagen, Hilden, Germany), with a total volume of 20 *μ*L. The cycle condition of PCR was 95°C for 2 minutes. This was followed by 11 cycles of 94°C for 20 seconds, 65°C for 40 seconds (minus 0.5°C for each cycle), and 72°C for 1.5 minutes. This was followed by 24 cycles, lasting for 20 seconds at 94°C, 30 seconds at 59°C, and 1.5 minutes at 72°C. Then 72°C for 2 minutes. The amplified samples were kept at 4°C. The sequence of SNaPshot multiple single-base extended reaction primers for each SNP is listed in Supplementary Table 1. The amplification reaction system was 10 *μ*L, including 2 *μ*L amplification product, 1 *μ*L primer with a final concentration of 0.8 mM, 5 *μ*L SNaPshot Multiplex kit (Applied Biosystems, Foster City, CA, USA), and 2 *μ*L ultrapure water. The cycle condition of PCR amplification system was 96°C for 1 minute, 96°C for 10 seconds, 55°C for 5 seconds, and 60°C for 30 seconds altogether in 28 cycles. The amplified product was purified with 1 U SAP 10 *μ*L, purified at 37°C for 1 hour and inactivated at 75°C for 15 minutes. After purification, 0.5 *μ*L extended product was mixed with 0.5 *μ*L Liz120 size standard, 9 *μ*L Hi-Di, and denaturated at 95°C for 5 minutes. The results were analyzed by ABI3130XL sequencer and GeneMapper 4.1 Software (Applied Biosystems, Co. Ltd., USA). PCR primer sequence and extension primer sequence were listed in Supplementary Table 1.

### 2.3. Statistical Methods

Statistical analysis was performed using SPSS (version 22.0, IBM SPSS Inc., Chicago, IL, USA). Independent-samples *T* test was used for the comparison of age and IOP between the two groups. The Hardy-Weinberg Equilibrium (HWE) test was performed in the control group for each SNP. The differences in allele frequency and sex between cases and controls were evaluated using the Chi-squared test or Fisher's exact test. Linkage disequilibrium (LD) and haplotype blocks were estimated using the Haploview 4.2 program [27]. PLINK software was used to construct haplotypes and estimate haplotype frequencies for both cases and controls. The haplotype block was defined by the confidence interval (CI) method implemented in the Haploview software. LD is represented by *D*′. Benjamin and Hochberg step-up false discovery rate (FDR) was used to correct multiple testing. The three standard genetic models were assumed: dominant, recessive, and additive. Corrected *P* (*P*_*c*_) < 0.05 was considered statistically significant. Odds ratio (OR) and 95% CI were estimated whenever applicable.

## 3. Results

### 3.1. Demographic and Clinical Features of Patients and Control Subjects

The mean age of the PSS group (83 males (55.3%) and 67 females (44.7%)) was 39.08 ± 12.51 years. The mean age of the control group (94 males (51.4%) and 89 females (48.6%)) was 41.19 ± 9.89 years. No significant difference in age and sex was found between the patient group and control group (*P* = 0.087 and 0.470, respectively, Table 1). The mean (SD) IOP of eyes with PSS was 41.20 ± 3.79 mmHg while 15.10 ± 2.59 mmHg in the control group. The IOP of eyes with PSS was significantly higher than that in healthy controls (*P* < 0.001, Table 1).

### 3.2. HLA-III Allele Frequencies in PSS Patients and Controls

The genotype distributions of *HLA-III* loci in control groups did not violate the HWE (all *P* > 0.05). Among the 21 SNPs, the frequencies of T allele at rs12190359 of *HSP70-1* and T allele at rs6457452 of *HSP70-2* in the patient group were significantly lower than those in healthy controls (0.33% vs. 3.28%, *P* = 0.006, and *P*_*c*_ = 0.018, OR = 0.10; 2.67% vs. 6.01%, *P* = 0.038, *P*_*c*_ = 0.076, and OR = 0.43; Table 2), and the frequencies of G allele at rs909253, A allele at rs1041981, G allele at rs2844484 of *TNF-β,* and A allele at rs1800629 of *TNF-α* in the patient group were significantly higher than those in healthy controls (62.00% vs. 53.01%, *P* = 0.020, *P*_*c*_ = 0.040, and OR = 1.45; 62.00% vs. 53.01%, *P* = 0.020, *P*_*c*_ = 0.033, and OR = 1.45; 76.67% vs. 67.49%, *P* = 0.009, *P*_*c*_ = 0.045, and OR = 1.58; 11.00% vs. 6.01%, *P* = 0.020, *P*_*c*_ = 0.100, and OR = 1.93; Table 2), but rs6457452 and rs1800629 did not survive the FDR correction. No significant difference in allele frequencies of other *HLA-III* SNPs was found between PSS patients and healthy controls (all *P* > 0.05, Table 2 and Supplementary Table 2).

### 3.3. HLA-III Haplotype Frequencies in PSS Patients and Controls

The frequencies of the CCT haplotype of *HSP70-1* (rs1008438-rs562047-rs12190359) and the ACCCTTT haplotype of *HSP70* (rs2227956-rs1043618-rs1008438-rs562047-rs12190359-rs2763979-rs6457452) were significantly lower in PSS patients than those in healthy controls (0.33% *vs.* 3.28%, *P* = 0.006, *P*_*c*_ = 0.024, and OR = 0.10; 0.34% *vs.* 3.32%, *P* = 0.006, *P*_*c*_ = 0.048, and OR = 0.10, respectively; Table 3). The TT haplotype of *HSP70-2* (rs2763979-rs6457452) and the GAAAC haplotype of *TNF-β* (rs2857709-rs2844484-rs909253-rs2229092-rs1041981) were significantly lower in PSS patients than those in healthy controls, while the frequencies of the TCCAG haplotype of *TNF-α* (rs1799964-rs1800630-rs1799724-rs1800629-rs361525) and the GGGAATCCAG haplotype of *TNF* (rs2857709-rs2844484-rs909253-rs2229092-rs1041981-rs1799964-rs1800630-rs1799724-rs1800629-rs361525) were significantly higher in PSS patients than those in healthy controls (2.67% *vs.* 6.01%, *P* = 0.038, *P*_*c*_ = 0.114, and OR = 0.43; 23.08% *vs.* 31.58%, *P* = 0.013, *P*_*c*_ = 0.052, and OR = 0.64; 11.11% *vs.* 6.09%, *P* = 0.020, *P*_*c*_ = 0.080, and OR = 1.93; 11.15% *vs.* 6.18%, *P* = 0.029, *P*_*c*_ = 0.174, and OR = 1.84, respectively; Table 3), but these haplotypes did not survive the FDR correction. No significant difference in haplotype frequencies of the other loci was found between patients with PSS and healthy controls (all *P* > 0.05, Supplementary Table 3).

### 3.4. Linkage Disequilibrium Analysis

**Figure 1:**
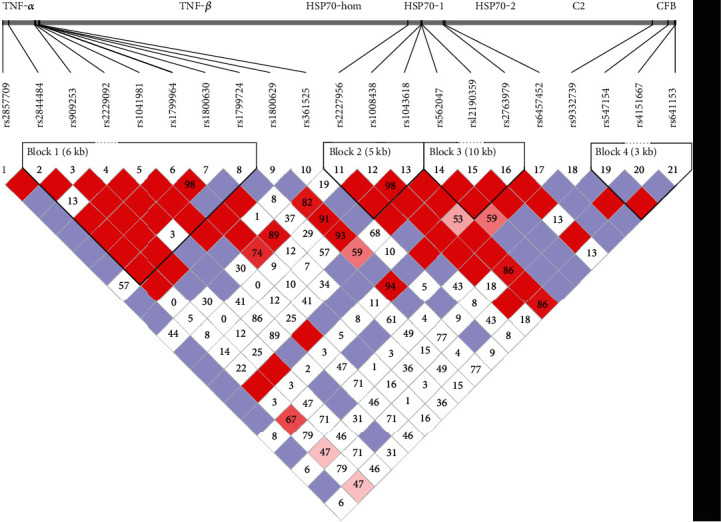
Pairwise LD (*D*′) among 21 SNPs in *HLA-III* genes.Pairs of D′ (× 100) values are displayed as blocks (no *D*′ values for 1.0 are displayed). The haplotype block was defined by the confidence interval method implemented in the Haploview software.

Strong LD was observed between the following SNPs (Figure 1): rs2227956 and rs1043618 of *HSP70-hom* (*D*′ = 1); rs1008438, rs562047, and rs12190359 of *HSP70-1* (*D*′ > 0.9); rs2763979 and rs6457452 of *HSP70-2* (*D*′ = 1); rs2857709, rs2844484, rs909253, rs1799964, rs1800630, rs1699964, and rs1041981 of *TNF-α* and *TNF-β* (*D*′ > 0.9); rs9332739, rs547154, rs4151667, and rs641153 of *C2* and *CFB* (*D*′ > 0.9). The weak LD was observed between rs2844484 and rs2229092 (*D*′ = 0.136), rs2229092 and rs1799724 (*D*′ = 0.036), and between rs2857709 and rs1799724 (*D*′ = 0.579).

### 3.5. Genotypic Association of HLA-III SNPs and PSS

To evaluate the genotypic association of the *HLA-III* SNPs and PSS, three different genetic models including dominant, recessive and additive model were applied. After FDR correction, we found that in additive model, rs1041981-AA and rs2844484-GG carriers showed nominally higher risk for PSS than rs1041981-CC and rs2844484-AA (*P*_*c*_ = 0.044, OR = 2.05, and 95% CI = 1.07 − 3.91 and *P*_*c*_ = 0.038, OR = 2.61, and 95% CI = 1.03 − 6.62, respectively; Supplementary Table 6). In addition, dominant model of rs12190359 and rs6457452 suggested that rs12190359-TT/CT and rs6457452-TT/CT carriers had decreased risks for PSS compared with rs12190359-CC and rs6457452-CC carriers (*P*_*c*_ = 0.006, OR = 0.10, and 95% CI = 0.01 − 0.74 and *P*_*c*_ = 0.018, OR = 0.36, and 95% CI = 0.15 − 0.86; Supplementary Table 4). No significant genotypic association was detected between the other SNPs and PSS (Supplementary Tables 4–6).

## 4. Discussion

In previous studies, we have shown the associations of *HLA-I* and *-II* polymorphisms with PSS at high resolution level in a southern Chinese Han population [10–12]. However, the associations between *HLA-III* gene polymorphisms and PSS have not been well evaluated. In the present study, 21 loci of 7 *HLA-III* genes were analyzed to further evaluate the associations between *HLA-III* gene polymorphisms and PSS in a southern Chinese Han population.

HSP70-1 played a cellular protective role as a guardian of lysosomal membrane integrity by assisting sphingomyelin degradation or by acting as a chaperone to maintain appropriate protein folding and cycling. HSP70-2 played an antiapoptotic role in neuron-trauma model [28]. In the present study, we for the first time found that the frequency of T allele at rs12190359 of *HSP70-1* in patients with PSS was significantly lower than that in healthy controls (Table 2), suggesting that T allele at rs12190359 might be a protective factor for PSS. The rs12190359 (amino acid 116) is the ATP binding site of HSP70 protein, and the cytosine changes to thymine can lead to the mutation of amino acid codon [29]. There was still no direct evidence that this mutation could change the protein content expression or function, so the possible relationship between PSS and changes caused by this mutation at rs12190359 requires further study. Temple et al. reported that the rs6457452 gene polymorphism at the promoter of *HSP70-2* was closely related to the expression of HSP70-1A/B [30]. Because the rs1061581 is a silent mutation, it is likely to be a surrogate marker for the rs6457452 in *HSP70-2* 50′-UTR, and the T allele at rs6457452 of *HSP70-2* reduced the HSP70 synthesis levels of normal-status cultured cells and the normal bronchial epithelium [31]. In the present study, we found that the frequency of T allele at rs6457452 of *HSP70-2* in patients with PSS was significantly lower than that in healthy controls (Table 2), suggesting that T allele at rs6457452 of *HSP70-2* might be a protective factor for PSS. We hypothesized that the amino acid altering from cytosine to thymine at rs6457452 of *HSP70-2* might affect the progression of PSS by affecting the expression level of HSP70. According to the dominant model of rs12190359 and rs6457452, rs12190359-TT/CT and rs6457452-TT/CT carriers had decreased risks for PSS compared with rs12190359-CC and rs6457452-CC carriers, indicating that T allele of rs12190359 and rs6457452 might be protective factors for PSS (Supplementary Table 4). In addition, we found the strong LD between *HSP70-1* (rs1008438-rs562047-rs12190359) and *HSP70-2* (rs2763979-rs6457452) (Figure 1). The frequency of CCT haplotype of *HSP70-1* (rs1008438-rs562047-rs12190359) was significantly lower in PSS patients than those in healthy controls (Table 3), suggesting that individuals carrying CCT haplotype of *HSP70-1* (rs1008438-rs562047-rs12190359) might decrease the risk of PSS. However, we did not find significant difference in allele frequencies of the *HSP70-hom* (rs1043618, rs2227956), *HSP70-1* (rs1008438, rs562047), and *HSP70-2* (rs2763979) loci between the PSS groups and healthy controls.

Several studies have shown that TNF plays an important role in the onset of uveitis and glaucoma [17–21, 32, 33]. Previous studies have shown that increased TNF-*α* production caused by the A allele at rs361525 in the promoter region of *TNF-α* was a susceptibility factor for Behcet's disease while the A/A genotype at rs1800629 of *TNF-α* had a significant protective effect on Behcet's disease [25, 32]. It was suggested by Xin et al. that the G/A genotype at rs1800629 of *TNF-α* gene was significantly associated with the risk of high-tension glaucoma in Asian populations [19]. A previous study by Bozkurt et al. suggested that the G/A genotype at rs1800629 of *TNF-α* gene might be related to primary open angle glaucoma in Turkish [26]. It has also been reported that allele A at rs1800629 of *TNF-α* gene was associated with increased TNF-*α* levels which might appear to influence CMV-induced pathology [33]. Polymorphisms of alleles A and G at rs1800629 in the promoter area of the gene coding for *TNF-a* are associated with an increased transcription activity of this gene, increased TNF-*α* production [26]. Despite *TNF-α* gene was associated with uveitis and glaucoma in various populations, the pathogenesis of PSS is different from glaucoma and uveitis to some extent, resulting there were no significant correlation between *TNF-α* gene polymorphism and PSS [25]. In the present study, we found that the A allele at rs1800629 of *TNF-α* might be the risk factor for PSS, indicating that A allele at rs1800629 of *TNF-α* might be associated with the pathogenesis of PSS induced by CMV infection. We also found that the A allele at rs1041981, A allele at rs909253, and G allele at rs2844484 of *TNF-β* might be risk factors for PSS. Previous studies have found that the C allele in exon 3 at rs1041981 of *TNF-β* tends to mutate to A, resulting in the mutation of threonine to aspartic acid [34]. Lu et al. observed that TNF-*β* levels produced were lower in peripheral blood mononuclear cells culture supernatants from allele A at rs909253 of *TNF-β* carrier compared to allele G homozygotes [35]. Individuals with a haplotype consisting of CC genotype of rs909253 and AA genotype of rs1041981 produced substantially higher amounts of TNF-*β* in their peripheral blood mononuclear cells [36]. We found that G allele at rs909253 and A allele at rs1041981 of *TNF-β* might be risk factors for PSS, indicating that the amino acid altering from threonine to asparagine at rs909253 and threonine to aspartic acid at rs1041981 of *TNF-β* might affect the progression of PSS by affecting the expression level of TNF-*β*. Thus, the individual SNPs may have minimal functional impact, and it is likely that rs909253 and rs1041981 of *TNF-β* affect the functional expression in linkage disequilibrium forming a functional haplotype. Besides, rs2844484 of *TNF-β* is positioned upstream from the start of *TNF-β,* and this SNP is part of a region identified as a hypersensitive site shown to contain upstream stimulatory factors [37]. This family of transcription factors has the capability to vary TNF-*β* expression when under stress and immune-related conditions. In the present study, we found that allele G at rs2844484 of *TNF-β* might be a risk factor for PSS, which might affect the progression of PSS by affecting the expression level of TNF-*β*. We also found that the rs1041981-AA and rs2844484-GG carriers showed nominally higher risk for PSS than rs1041981-CC and rs2844484-AA (Supplementary Table 6). Furthermore, we found that the GAAAC haplotype of *TNF-β* gene (rs2857709-rs2844484-rs909253-rs2229092-rs1041981) might be a protective factor, while the GGGAATCCAG haplotype of *TNF* gene (rs2857709-rs2844484-rs909253-rs2229092-rs1041981-rs1799964-rs1800630-rs1799724-rs1800629-rs361525) might be a risk factor for PSS in a southern Han Chinese population. However, we found no significant difference in allele frequencies of the *TNF-α* (rs361525, rs1799964, rs1799724, and rs1800630) and *TNF-β* (rs2857709 and rs2229092) loci between PSS patients and healthy controls (Table 2).

In addition, we did not find significant difference in allele frequencies of *CFB* (rs641153 and rs4151667) and *C2* (rs9332739 and rs547154) between PSS patients and controls. Our results suggested that these complement factors of *HLA-III* genes might not be related to the development of PSS in southern Chinese. Due to the large number of genes in the complement family, we analyzed these SNPs located in chromosome 6 and did not find significant association between these SNPs and PSS. However, there were some limitations in this study. The LD region of 6p21.3 is extensive and contains a lot of transcripts, dissection, and elucidation of the association between *HLA-III* polymorphisms and PSS requires additional investigations with larger sample sizes in different ethnic groups. We only evaluated the associations between *HLA-III* gene polymorphisms and PSS patients in a southern Chinese Han population. The geographical scope and the number of patients with PSS were limited, and all loci included in the *HLA-III* gene were not completely sequenced. Our team will continue to collect data from different regions and further investigate the association between PSS patients in other regions and *HLA-III* gene polymorphisms. Other genes will also be investigated to further elucidate the pathogenesis of PSS.

## 5. Conclusion

In summary, the G allele at rs909253, A allele at rs1041981, and G allele at rs2844484 of *TNF-β* gene might be risk factors for PSS, while the T allele at rs12190359 of *HSP70-1* gene, the CCT haplotype of *HSP70-1* gene (rs1008438-rs562047-rs12190359), and the ACCCTTT haplotype of *HSP70* gene (rs2227956-rs1043618-rs1008438-rs562047-rs12190359-rs2763979-rs6457452) might be protective factors for PSS in southern Chinese. These findings can provide valuable new clues for investigation into the mechanisms and development of new diagnosis and treatment for PSS.

## Figures and Tables

**Table 1:** The demographic and clinical features of the PSS cases and controls.

Feature	PSS (*n* = 150)	Control (*n* = 183)	*P*
Sex(M/F)	83/67	94/89	0.470^a^
Age (year, mean ± SD)	39.08 ± 12.51	41.19 ± 9.89	0.087^b^
IOP (mmHg, mean ± SD)	41.20 ± 3.79	15.10 ± 2.59	< 0.000^b^
KPs (*Y*/*N*)	*Y*	*N*	

n: number of subjects; PSS: Posner-Schlossman syndrome; *P*: *P* value; IOP: intraocular pressure; KPs: keratic precipitates; ^a^: Chi-squared test; ^b^: independent-samples *T* test.

**Table 2:** *HLA-III* allele frequencies in PSS cases and controls.

Gene	Variants	Location region	Functional annotation	Allele	PSS (2*n* = 300)	Control (2*n* = 366)	*P*	*P*_*c*_	OR (95% CI)
*HSP70-1*	rs1008438	Upstream	TFBS	C	119 (39.67)	145 (39.62)	0.990	0.990	1.00 (0.73-1.37)
				A	181 (60.33)	221 (60.38)			
	rs562047	Exonic (nonsynonymous)	nsSNP	C	31 (10.33)	37 (10.11)	0.924	1.000	1.03 (0.62-1.70)
				G	269 (89.67)	329 (89.89)			
	rs12190359	Exonic (nonsynonymous)	TFBS	T	1 (0.33)	12 (3.28)	**0.006**	**0.018**	0.10 (0.01-0.76)
				C	299 (99.67)	354 (96.72)			

*HSP70-2*	rs2763979	Upstream	TFBS	T	72 (24.00)	91 (24.86)	0.797	0.797	1.05 (0.73-1.50)
				C	228 (76.00)	275 (75.14)			
	rs6457452	5′UTR	TFBS	T	8 (2.67)	22 (6.01)	**0.038**	0.076	0.43 (0.19-0.98)
				C	292 (97.33)	344 (93.99)			

*TNF-α*	rs361525	Upstream	TFBS	A	3 (1.00)	4 (1.09)	0.860	0.860	1.09 (0.24-4.93)
				G	297 (99.00)	362 (98.91)			
	rs1800629	Intergenic	TFBS	A	33 (11.00)	22 (6.01)	**0.020**	0.100	1.93 (1.10-3.39)
				G	267 (89.00)	344 (93.99)			
	rs1799724	Intergenic	TFBS	T	29 (9.67)	45 (12.30)	0.283	0.708	1.31 (0.80-2.15)
				C	271 (90.33)	321 (87.70)			
	rs1799964	Downstream	TFBS	C	44 (14.67)	52 (14.21)	0.867	1.000	1.04 (0.67-1.60)
				T	256 (85.33)	314 (85.79)			
	rs1800630	Intergenic	/	A	42 (14.00)	49 (13.39)	0.819	1.000	1.05 (0.68-1.64)
				C	258 (86.00)	317 (86.61)			

*TNF-β*	rs909253	Intronic	TFBS	A	114 (38.00)	172 (46.99)	**0.020**	**0.040**	1.45 (1.06-1.97)
				G	186 (62.00)	194 (53.01)			
	rs1041981	Exonic (nonsynonymous)	ESE or ESS, nsSNP	C	114 (38.00)	172 (46.99)	**0.020**	**0.033**	1.45 (1.06-1.97)
				A	186 (62.00)	194 (53.01)			
	rs2857709	Intronic	/	A	1 (0.33)	5 (1.37)	0.230	0.328	4.14 (0.48-35.64)
					299 (99.67)	361 (98.63)			
	rs2844484	Intronic	TFBS	A	70 (23.33)	119 (32.51)	**0.009**	**0.045**	1.58 (1.12-2.24)
				G	230 (76.67)	247 (67.49)			
	rs2229092	Exonic (nonsynonymous)	ESE or ESS, nsSNP	C	2 (0.67)	6 (1.64)	0.305	0.305	2.48 (0.50-12.39)
				A	298 (99.33)	360 (98.36)			

The allele frequencies were presented as allele count (%). *P* value was calculated using Chi-squared test or Fisher's exact test and corrected for multiple testing using the FDR method. *n*: number of subjects; PSS: Posner-Schlossman syndrome; *P*: *P* value; *P_c_*: corrected *P* value; CI: confidence interval; OR: odds ratio; TFBS: transcription factor binding sites; ESE: exon splicing enhancer; ESS: exon splicing silencer; nsSNP: nonsynonymous single nucleotide polymorphism. *P* values less than 0.05 are bolded.

**Table 3:** *HLA-III* haplotype frequencies between PSS patients and healthy controls.

	Frequency (%)		*P*	*P*_*c*_	OR (95% CI)
	PSS (2*n* = 300)	Control (2*n* = 366)			
*HSP70-1* rs1008438-rs562047-rs12190359					
CCT	0.33	3.28	**0.006**	**0.024**	0.10 (0.01-0.76)
CCC	10	6.83	0.139	0.278	1.52 (0.87-2.64)
CGC	29.33	29.51	0.961	1.000	0.99 (0.71-1.39)
AGC	60.330	60.38	0.990	0.990	1.00 (0.73-1.36)
*HSP70-2* rs2763979-rs6457452					
TT	2.67	6.01	**0.038**	0.114	0.43 (0.19-0.98)
TC	21.33	18.85	0.426	0.639	1.17 (0.80-1.71)
CC	76.00	75.14	0.797	0.797	1.05 (0.73-1.50)
*HSP70* rs2227956-rs1043618-rs1008438-rs562047-rs12190359-rs2763979-rs6457452					
ACCCTTT	0.34	3.32	**0.006**	**0.048**	0.10 (0.01-0.76)
ACCGCCC	15.31	14.90	0.913	0.913	1.02 (0.67-1.57)
ACCGCTC	5.56	4.71	0.551	1.000	1.23 (0.62-2.46)
AGAGCCC	38.14	36.47	0.659	1.000	1.07 (0.78-1.47)
ACCCCTC	9.10	6.09	0.142	0.568	1.55 (0.86-2.78)
GGAGCCC	23.16	24.31	0.691	0.921	0.93 (0.65-1.33)
AGCGCTC	7.14	8.27	0.563	1.000	0.84 (0.47-1.51)
ACCGCTT	1.26	1.93	0.762	0.871	0.69 (0.20-2.39)
*TNF-α* rs1799964-rs1800630-rs1799724-rs1800629-rs361525					
TCCAG	11.11	6.09	**0.020**	0.080	1.93 (1.10-3.39)
TCTGG	9.43	12.46	0.186	0.372	0.72 (0.44-1.18)
CACGG	13.80	13.30	0.917	0.917	1.02 (0.66-1.60)
TCCGG	65.66	68.15	0.518	0.691	0.90 (0.65-1.24)
*TNF-β* rs2857709-rs2844484-rs909253-rs2229092-rs1041981					
GGACC	0.67	1.66	0.305	0.407	0.40 (0.08-2.01)
GAAAC	23.08	31.58	**0.013**	0.052	0.64 (0.46-0.91)
GGAAC	14.05	13.02	0.740	0.740	1.08 (0.69-1.68)
GGGAA	62.21	53.74	**0.027**	0.054	1.42 (1.04-1.94)
*TNF* rs2857709-rs2844484-rs909253-rs2229092-rs1041981-rs1799964-rs1800630-rs1799724-rs1800629-rs361525					
GAAACTCCGG	13.85	19.38	0.065	0.195	0.68 (0.45-1.03)
GGGAATCCGG	51.69	48.31	0.396	0.475	1.14 (0.84-1.55)
GGGAATCCAG	11.15	6.18	**0.029**	0.174	1.84 (1.06-3.21)
GGAACCACGG	13.18	11.80	0.538	0.538	1.16 (0.73-1.83)
GAAACTCTGG	9.46	12.64	0.186	0.372	0.72 (0.44-1.18)
GGACCCACGG	0.68	1.69	0.252	0.378	0.40 (0.08-2.01)

The haplotype frequencies were presented as haplotype ratio (%). *P* value was calculated using Chi-squared test or Fisher's exact test and corrected for multiple testing using the FDR method. *n*: number of subjects; PSS: Posner-Schlossman syndrome; *P*: *P* value; *P*_*c*_: corrected *P* value; CI: confidence interval; OR: odds ratio; *P* values less than 0.05 are bolded.

## Data Availability

All relevant data is within the paper. All raw data remains in the possession of the authors of the article.

## Supplementary Materials

Supplementary Materials Supplementary Table 1: characteristic information, product size, and primers of the SNPs in *HLA-III* genes. Supplementary Table 2: other *HLA-III* allele frequencies in PSS cases and controls. Supplementary Table 3: other *HLA-III* haplotype frequencies between PSS patients and healthy controls. Supplementary Table 4: dominant genetic models of *HLA-III* gene in PSS cases and controls. Supplementary Table 5: recessive genetic models of *HLA-III* gene in PSS cases and controls. Supplementary Table 6: additive genetic models of *HLA-III* gene in PSS cases and controls. *(Supplementary Materials)*

## References

[1] Posner A., Schlossman A. (1948). Syndrome of unilateral recurrent attacks of glaucoma with cyclitic symptoms. *Archives of Ophthalmology*.

[2] Megaw R., Agarwal P. K. (2017). Posner-Schlossman syndrome. *Survey of Ophthalmology*.

[3] Hedayatfar A., Chee S. P. (2014). Posner-Schlossman syndrome associated with cytomegalovirus infection: a case series from a non-endemic area. *International Ophthalmology*.

[4] Shen S. C., Ho W. J., Wu S. C. (2010). Peripheral vascular endothelial dysfunction in glaucomatocyclitic crisis: a preliminary study. *Investigative Ophthalmology & Visual Science*.

[5] Chee S. P., Jap A. (2008). Presumed fuchs heterochromic iridocyclitis and Posner-Schlossman syndrome: comparison of cytomegalovirus-positive and negative eyes. *American journal of ophthalmology*.

[6] Cao G., Tan C., Zhang Y. (2019). Digital droplet polymerase chain reaction analysis of common viruses in the aqueous humour of patients with Posner-Schlossman syndrome in Chinese population. *Clinical & Experimental Ophthalmology*.

[7] Chen W., Zhao J., Zhu T. (2017). Detection and analysis on serum antibodies for five common pathogenic microbes in patients with Posner-Schlossman syndrome. *Chinese Journal of Experimental Ophthalmology*.

[8] Dendrou C. A., Petersen J., Rossjohn J., Fugger L. (2018). HLA variation and disease. *Nature Reviews Immunology*.

[9] Hirose S., Ohno S., Matsuda H. (1985). HLA-Bw54 and glaucomatocyclitic crisis. *Archives of Ophthalmology*.

[10] Zhao J., Zhu T., Chen W. (2015). Human leukocyte antigens-B and -C loci associated with Posner-Schlossman syndrome in a southern Chinese population. *PLoS One*.

[11] Zhao J., Zhu T., He L., Shen X., Wang Y., Deng Z. (2015). Association of HLA-DPA1 and -DPB1 polymorphisms with Posner-Schlossman syndrome among southern Chinese Han population. *Journal of Medical Genetics*.

[12] Huang X., Xu Y., Chen W. (2019). The genetic contribution of HLA-E 01:03 and HLA-E 01:03-G 01:01 to Posner-Schlossman syndrome in southern Chinese. *Annals of Translational Medicine*.

[13] Medhasi S., Chantratita N. (2022). Human leukocyte antigen (HLA) system: genetics and association with bacterial and viral infections. *Journal of Immunology Research*.

[14] Radons J. (2016). The human HSP70 family of chaperones: where do we stand. *Cell Stress & Chaperones*.

[15] Ayub H., Khan M. I., Micheal S. (2010). Association of eNOS and HSP70 gene polymorphisms with glaucoma in Pakistani cohorts. *Molecular Vision*.

[16] Salehi Z., Gholaminia M., Gholaminia Z., Panjtanpanah M., Qazvini M. G. (2017). The GG genotype of the HSPA1B gene is associated with increased risk of glaucoma in northern Iran. *Molekuliarnaia Biologiia (Mosk)*.

[17] Tezel G., Li L. Y., Patil R. V., Wax M. B. (2001). TNF-alpha and TNF-alpha receptor-1 in the retina of normal and glaucomatous eyes. *Investigative Ophthalmology & Visual Science*.

[18] Liu B., Sen H. N., Nussenblatt R. (2012). Susceptibility genes and pharmacogenetics in ocular inflammatory disorders. *Ocular Immunology and Inflammation*.

[19] Xin X., Gao L., Wu T., Sun F. (2013). Roles of tumor necrosis factor alpha gene polymorphisms, tumor necrosis factor alpha level in aqueous humor, and the risks of open angle glaucoma: a meta-analysis. *Molecular Vision*.

[20] Wang C. Y., Shen Y. C., Wei L. C. (2012). Polymorphism in the TNF-*α* (-863) locus associated with reduced risk of primary open angle glaucoma. *Molecular Vision*.

[21] Khera T. K., Dick A. D., Nicholson L. B. (2010). Mechanisms of TNF-*α* regulation in uveitis: focus on RNA-binding proteins. *Progress in Retinal and Eye Research*.

[22] de Medeiros F. A., Alfieri D. F., Iriyoda T. M. (2019). TNF-*β* +252 A>G (rs909253) polymorphism is independently associated with presence of autoantibodies in rheumatoid arthritis patients. *Clinical and Experimental Medicine*.

[23] Chen W., Zhao J., Zhu T., Peng S., Huang X. (2016). Activation of serum complement in Posner-Schlossman syndrome patients. *Chinese Journal of Experimental Ophthalmology*.

[24] Huang X., Liu X., Ye Y. (2021). Polymorphisms and circulating plasma protein levels of immune checkpoints (CTLA-4 and PD-1) are associated with Posner-Schlossman syndrome in southern Chinese. *Frontiers in Immunology*.

[25] Bonyadi M. H. J., Yaseri M., Soheilian M. (2020). Tumor necrosis factor (TNF)-308, -1031, and angiotensin-converting enzyme (ACE) DD/II polymorphisms' role in Behcet's disease with and without uveitis: a meta-analysis. *Ophthalmic Genetics*.

[26] Bozkurt B., Mesci L., Irkec M. (2012). Association of tumour necrosis factor-alpha -308 G/A polymorphism with primary open-angle glaucoma. *Clinical & Experimental Ophthalmology*.

[27] Slatkin M. (2008). Linkage disequilibrium--understanding the evolutionary past and mapping the medical future. *Nature Reviews Genetics*.

[28] Fang C. T., Kuo H. H., Pan T. S., Yu F. C., Yih L. H. (2016). HSP70 regulates the function of mitotic centrosomes. *Cellular and Molecular Life Sciences*.

[29] Zhang X. W., Li Y. P., Shi L. (2016). The association of SNPS in HSPA1A gene with lung cancer in Yunnan Han population. *Journal of GuiZhou medical university*.

[30] Temple S. E., Cheong K. Y., Ardlie K. G., Sayer D., Waterer G. W. (2004). The septic shock associated HSPA1B1267 polymorphism influences production of HSPA1A and HSPA1B. *Intensive Care Medicine*.

[31] Guo H., Deng Q., Wu C. (2011). Variations in HSPA1B at 6p21.3 are associated with lung cancer risk and prognosis in Chinese populations. *Cancer Research*.

[32] Higuchi T., Seki N., Kamizono S. (1998). Polymorphism of the 5'-flanking region of the human tumor necrosis factor (TNF)-alpha gene in Japanese. *Tissue Antigens*.

[33] Edwar L., Ha P., Ariyanto I. A. (2021). A TNF block genotype may influence CMV retinitis in HIV patients without affecting systemic viral replication. *Current HIV Research*.

[34] Kádár K., Kovács M., Karádi I. (2008). Polymorphisms of TNF-alpha and LT-alpha genes in multiple myeloma. *Leukemia Research*.

[35] Lu L. Y., Cheng H. H., Sung P. K., Tai M. H., Yeh J. J., Chen A. (2005). Tumor necrosis factor-beta +252A polymorphism is associated with systemic lupus erythematosus in Taiwan. *Journal of the Formosan Medical Association*.

[36] Messer G., Spengler U., Jung M. C. (1991). Polymorphic structure of the tumor necrosis factor (TNF) locus: an NcoI polymorphism in the first intron of the human TNF-beta gene correlates with a variant amino acid in position 26 and a reduced level of TNF-beta production. *The Journal of Experimental Medicine*.

[37] Taylor J. M., Wicks K., Vandiedonck C., Knight J. C. (2008). Chromatin profiling across the human tumour necrosis factor gene locus reveals a complex, cell type-specific landscape with novel regulatory elements. *Nucleic Acids Research*.

