# Supplementary material for: HSP70 and TNF Loci Polymorphism Associated with the Posner-Schlossman Syndrome in a Southern Chinese Population
Source: J Immunol Res. 2022 Dec 9;2022:5242948. doi: 10.1155/2022/5242948 (PMC9757935; doi:10.1155/2022/5242948)
Supplement: Supplementary Materials — Supplementary Table 1: characteristic information, product size, and primers of the SNPs in HLA-III genes. Supplementary Table 2: other HLA-III allele frequencies in PSS cases and controls. Supplementary Table 3: other HLA-III haplotype frequencies between PSS patients and healthy controls. Supplementary Table 4: dominant genetic models of HLA-III gene in PSS cases and controls. Supplementary Table 5: recessive genetic models of HLA-III gene in PSS cases and controls. Supplementary Table 6: additive genetic models of HLA-III gene in PSS cases and controls. (Supplementary Materials) [file 5242948.f1.zip › Supplementary Table 1 (2).docx]

# Supplementary Table 1. Characteristic information, product size and primers of the SNPs in *HLA-Ⅲ* genes

| SNP ID | Chromosome | Position | Mutation | Product size (bp) | PCR primer sequence | Extension primer sequence |
| --- | --- | --- | --- | --- | --- | --- |
| *TNF-β* |  |  |  |  |  |  |
| rs2857709 | 6 | 31532814 | G>A | 179 | F: GATGATGGTAGGGATAAGATGTGGAGA  R: TTTTCTCTCCCCCAGACCACTG | SF: TTTTTTTTTTTTTTTGATTGTGCTGTGAAGACAGGAA |
| rs2844484 | 6 | 31536224 | G>A | 226 | F: AAACAGGGTTGGGTTGGGGTAG  R: TGAAGCCTCCAAATGAGACTGAAA | SF: TTTTTTTTTTTTTTTTTTGGTTATTAATTGGGATGTGTTTAGATTT |
| rs909253 | 6 | 31540313 | G>A | 723 | F: CGTGCTTCGTGCTTTGGACTAC  R: GTCTGGGAGGTCAGGTGGATGT | SR: TTTTTTTTTTTTTTTTTTTTCAGGAAGGGAACAGAGAGGAA |
| rs2229092 | 6 | 31540757 | A>C | 723 | F: CGTGCTTCGTGCTTTGGACTAC  R: GTCTGGGAGGTCAGGTGGATGT | SF: TTTTTTTTTTTTTTTTTTTTTTTTTTTTTTTTTTTTTCCCAGACTGCCCGTCAGC |
| rs1041981 | 6 | 31540784 | A>C | 723 | F: CGTGCTTCGTGCTTTGGACTAC  R: GTCTGGGAGGTCAGGTGGATGT | SF: TTTTTTTTTTTTTTTTTTTTTTTTTTTTTTTTATGCATCTTGCCCACAGCA |
| *TNF-α* |  |  |  |  |  |  |
| rs1799964 | 6 | 31542308 | T>C | 343 | F: GCTTCAGGGATATGTGATGGACTCA  R: CCATATCTTCTTAAACGTCCCCTGTATTC | SR: TTTTTTTTTTTTTTTTTCCAGACCCTGACTTTTCCTTC |
| rs1800630 | 6 | 31542476 | C>A | 343 | F: GCTTCAGGGATATGTGATGGACTCA  R: CCATATCTTCTTAAACGTCCCCTGTATTC | SR: TTTTTTTTTTTTTTTTTTTTTTTTTTTTTTTTCATGGCCCTGTCTTCRTTAAG |
| rs1800629 | 6 | 31543031 | G>A | 183 | F: CACAGACCTGGTCCCCAAAAGA  R: TGGAAAGTTGGGGACACACAAG | SR: TTTTTTTTTTTTTTTTTTTTGGAGGCTGAACCCCGTCC |
| rs361525 | 6 | 31542482 | C>T | 183 | F: CACAGACCTGGTCCCCAAAAGA  R: TGGAAAGTTGGGGACACACAAG | SR: TTTTTTTTTTTTTTTTCTCCCCATCCTCCCTGCTC |
| rs1799724 | 6 | 31543101 | G>A | 343 | F: GCTTCAGGGATATGTGATGGACTCA  R: CCATATCTTCTTAAACGTCCCCTGTATTC | SF: TTTTTTTTTTTTTTTTATGGGGACCCCCMCTTAA |
| *HSP70-hom* |  |  |  |  |  |  |
| rs1043618 | 6 | 31783208 | C>T | 959 | F: GCAAGCCCCCACAATTAAAAGC  R: CTTTGGCCATGCCGGTTC | SR: TTTTTTTTTTTTTTTTTTTTTTTTTTTTTTTTTTTTTTTTTTTTTTTTTTTCCCTGCTCTCTGTCGGCTC |
| rs2227956 | 6 | 31783863 | G>C | 182 | F: TTGCAGCAATTTTCTCCCTCTG  R: TGAYATTGATGCCAATGGTATTCTCA | SF: TTTTTTTTTTTTCTTGCCGGTGCTCTTGTCC |
| *HSP70-1* |  |  |  |  |  |  |
| rs1008438 | 6 | 31783881 | C>T | 959 | F: GCAAGCCCCCACAATTAAAAGC  R: CTTTGGCCATGCCGGTTC | SF: TTTTTTTTTTTTTTTTTTTTTTTTTTTTTTTGGCAGGACGGGAGGCGAAA |
| rs562047 | 6 | 31794592 | C>T | 959 | F: CTTCTCGCGGATCCAGTGTTC  R: CTCGGCGATCTCCTTCATCTTG | SF: TTTTTTTTTTTTTTTTTTTTTTTTTTTTTTTTTTTCAGGTGAGCTACAAGGGGGA |
| rs12190359 | 6 | 31795550 | C>T | 959 | F: CTTCTCGCGGATCCAGTGTTC  R: CTCGGCGATCTCCTTCATCTTG | SR: TTTTTTTTTTTTTTTTTTTTTTCACCATGGACGAGATCTCCTC |
| *HSP70-2* |  |  |  |  |  |  |
| rs2763979 | 6 | 31778272 | A>G | 206 | F: CTSCCAGGGTCACCATCTTGTT  R: GGCATTTGGGATTCCTCCATTT | SF: TTTTTTTTTTTTTTTTTGGACTGTGAGGTCCTACTTCTACACAC |
| rs6457452 | 6 | 31783507 | G>C | 577 | F: GCCAGCCTGAGGAGCTGCT  R: GACAAGAGCTCAGTCCTTCGGAAC | SR: TTTTTTTTTTTTTTTTTTTTTTTTTTTTTTTTTTTTTTTTTTTTTTTTTTTTGGAGTCACTCTSGAAAGACGAA |
| *C2* |  |  |  |  |  |  |
| rs9332739 | 6 | 31903804 | G>C | 217 | F: TACCATCTCCCCTTTGGCTTCA  R: TCACGTGATGACACCCGTACCT | SR: TTTTTTTTTTTTTTTTTTTTTTTTTTTTTTTTTTTTTTTTTTTTTTTTTTCCAGGCTGCTGATCAC |
| rs547154 | 6 | 31910938 | G>C | 243 | F: GCCTTCATTCTGCAGGACACAA  R: GGTCAGAGGCGTTTGCTGACAT | SR: TTTTTTTTTTTTTTTTTTTTTTTTTTTTTTTTTTTTTTTCACTGTGTCCAGGTTCCCAA |
| *CFB* |  |  |  |  |  |  |
| rs4151667 | 6 | 31914024 | T>A | 259 | F: GCCATGGGGAGCAATCTCAG  R: CCTGGCCCTCTTGGAGAAGTC | SR: TTTTTTTTTTTTTTTTTTTTTTTTTTTTTTTTTTTTTTTAAGATAAAGGGCATCAGGCAG |
| rs641153 | 6 | 31914180 | G>A | 259 | F: GCCATGGGGAGCAATCTCAG  R: CCTGGCCCTCTTGGAGAAGTC | SR: TTTTTTTTTTTTTTTTTTTTTTTTTTTTTTTTTTTTTTTTTTTTTAGAGCAGGATCCCTGGGGC |

SNPs information obtained from GRCh38; SNP: single nucleotide polymorphism; F, forward; R, reverse.
