# Supplementary material for: HSP70 and TNF Loci Polymorphism Associated with the Posner-Schlossman Syndrome in a Southern Chinese Population
Source: J Immunol Res. 2022 Dec 9;2022:5242948. doi: 10.1155/2022/5242948 (PMC9757935; doi:10.1155/2022/5242948)
Supplement: Supplementary Materials — Supplementary Table 1: characteristic information, product size, and primers of the SNPs in HLA-III genes. Supplementary Table 2: other HLA-III allele frequencies in PSS cases and controls. Supplementary Table 3: other HLA-III haplotype frequencies between PSS patients and healthy controls. Supplementary Table 4: dominant genetic models of HLA-III gene in PSS cases and controls. Supplementary Table 5: recessive genetic models of HLA-III gene in PSS cases and controls. Supplementary Table 6: additive genetic models of HLA-III gene in PSS cases and controls. (Supplementary Materials) [file 5242948.f1.zip › Supplementary Table 2 (2).docx]

**Supplementary Table 2.** **Other** ***HLA-Ⅲ* allele frequencies in PSS cases and controls**

| Gene | Variants | Annotation | Functional annotation | Allele | PSS  (2*n =* 300) | Control  (2*n =* 366) | *P* | *P_c_* | *OR* (*95% CI*) |
| --- | --- | --- | --- | --- | --- | --- | --- | --- | --- |
| *HSP70-hom* | rs1043618 | 5’UTR | TFBS | C | 97 (32.33) | 116 (31.69) | 0.86 | 0.86 | 1.03 (0.74-1.43) |
|  |  |  |  | G | 203 (67.67) | 250 (68.31) | 0.86 | 1 | 0.97 (0.70-1.35) |
|  | rs2227956 | exonic (nonsynonymous) | nsSNP | G | 68 (22.67) | 88 (24.04) | 0.676 | 1 | 0.93 (0.65-1.33) |
|  |  |  |  | A | 232 (77.33) | 278 (75.96) | 0.676 | 1 | 1.08 (0.75-1.55) |
| *CFB* | rs641153 | exonic (nonsynonymous) | ESE or ESS, nsSNP | A | 13 (4.33) | 23 (6.28) | 0.268 | 0.536 | 0.68 (0.34-1.36) |
|  |  |  |  | G | 287 (95.67) | 343 (93.72) | 0.268 | 1 | 1.48 (0.74-2.98) |
|  | rs4151667 | exonic (nonsynonymous) | ESE or ESS, nsSNP | A | 4 (1.33) | 6 (1.64) | 1 | 1 | 0.81 (0.23-2.90) |
|  |  |  |  | T | 296 (98.67) | 360 (98.36) | 1 | 1 | 1.23 (0.35-4.41) |
| *C2* | rs9332739 | exonic (nonsynonymous) | ESE or ESS, nsSNP | C | 4 (1.33) | 6 (1.64) | 1 | 1 | 0.81 (0.23-2.90) |
|  |  |  |  | G | 296 (98.67) | 360 (98.36) | 1 | 1 | 1.23 (0.35-4.41) |
|  | rs547154 | intronic | TFBS | T | 13 (4.33) | 23 (6.28) | 0.268 | 0.536 | 0.68 (0.34-1.36) |
|  |  |  |  | G | 287 (95.67) | 343 (93.72) | 0.268 | 1 | 1.48 (0.74-2.98) |

The allele frequencies were presented as allele count (%). *P* value was calculated using chi-squared test or Fisher’s exact test and corrected for multiple testing using the FDR method. n: number of subjects; PSS: Posner-Schlossman syndrome; *P*: *P* value; *P_c_*: corrected *P* value; *CI*: confidence interval; *OR*: odds ratio; TFBS: transcription factor binding sites; ESE: exon splicing enhancer; ESS: exon splicing silencer; nsSNP: nonsynonymous single nucleotide polymorphism.
