# Supplementary material for: HSP70 and TNF Loci Polymorphism Associated with the Posner-Schlossman Syndrome in a Southern Chinese Population
Source: J Immunol Res. 2022 Dec 9;2022:5242948. doi: 10.1155/2022/5242948 (PMC9757935; doi:10.1155/2022/5242948)
Supplement: Supplementary Materials — Supplementary Table 1: characteristic information, product size, and primers of the SNPs in HLA-III genes. Supplementary Table 2: other HLA-III allele frequencies in PSS cases and controls. Supplementary Table 3: other HLA-III haplotype frequencies between PSS patients and healthy controls. Supplementary Table 4: dominant genetic models of HLA-III gene in PSS cases and controls. Supplementary Table 5: recessive genetic models of HLA-III gene in PSS cases and controls. Supplementary Table 6: additive genetic models of HLA-III gene in PSS cases and controls. (Supplementary Materials) [file 5242948.f1.zip › Supplementary Table 3 (1).docx]

**Supplementary Table 3. Other *HLA-Ⅲ* haplotype frequencies between PSS patients and healthy controls**

|  | Frequency (%) | | *P* | *P_c_* | *OR* (*95%CI)* |
| --- | --- | --- | --- | --- | --- |
|  | PSS (2n=300) | Control (2n=366) |  |  |  |
| *HSP70-hom* rs2227956-rs1043681 | | | | | |
| AC | 32.33 | 31.69 | 0.860 | 0.860 | 1.03 (0.74-1.43) |
| GG | 22.67 | 24.04 | 0.676 | 1.000 | 0.93 (0.65-1.33) |
| AG | 45.00 | 44.26 | 0.849 | 1.000 | 1.03 (0.76-1.40) |
| *C2* rs9332739-rs547154 | | | | | |
| GT | 4.33 | 6.28 | 0.268 | 0.402 | 0.68 (0.34-1.36) |
| CG | 1.33 | 1.64 | 1.000 | 1.000 | 0.81 (0.23-2.90) |
| GG | 94.33 | 92.08 | 0.253 | 0.759 | 1.43 (0.77-2.66) |
| *CFB* rs4151667-rs641153 | | | | | |
| TA | 4.33 | 6.28 | 0.268 | 0.402 | 0.68 (0.34-1.36) |
| AG | 1.33 | 1.64 | 1.000 | 1.000 | 0.81 (0.23-2.90) |
| TG | 94.33 | 92.08 | 0.253 | 0.759 | 1.43 (0.77-2.66) |

The haplotype frequencies were presented as haplotype ratio (%). *P* value was calculated using chi-squared test or Fisher’s exact test and corrected for multiple testing using the FDR method. n: number of subjects; PSS: Posner-Schlossman syndrome; *P*: *P* value; *P_c_*: corrected *P* value; *CI*: confidence interval; *OR*: odds ratio.
