# Supplementary material for: HSP70 and TNF Loci Polymorphism Associated with the Posner-Schlossman Syndrome in a Southern Chinese Population
Source: J Immunol Res. 2022 Dec 9;2022:5242948. doi: 10.1155/2022/5242948 (PMC9757935; doi:10.1155/2022/5242948)
Supplement: Supplementary Materials — Supplementary Table 1: characteristic information, product size, and primers of the SNPs in HLA-III genes. Supplementary Table 2: other HLA-III allele frequencies in PSS cases and controls. Supplementary Table 3: other HLA-III haplotype frequencies between PSS patients and healthy controls. Supplementary Table 4: dominant genetic models of HLA-III gene in PSS cases and controls. Supplementary Table 5: recessive genetic models of HLA-III gene in PSS cases and controls. Supplementary Table 6: additive genetic models of HLA-III gene in PSS cases and controls. (Supplementary Materials) [file 5242948.f1.zip › Supplementary Table 4 (1).docx]

**Supplementary Table 4. Dominant genetic models of *HLA-Ⅲ* gene in PSS cases and controls**

| Gene | Variants | Dominant | PSS | Control | *P* | *P*_c_ | *OR (95%CI)* |
| --- | --- | --- | --- | --- | --- | --- | --- |
| *HSP70-1* | rs1008438 | CC+CA vs. AA | 96/54 | 115/68 | 0.827 | 1.000 | 1.05 (0.67-1.65) |
|  | rs562047 | CC+CG vs. GG | 30/120 | 37/146 | 0.961 | 0.961 | 0.99 (0.58-1.69) |
|  | rs12190359 | TT+CT vs. CC | 1/149 | 12/171 | **0.006** | **0.018** | 0.10 (0.01-0.74) |
| *HSP70-2* | rs2763979 | TT+CT vs. CC | 65/85 | 84/99 | 0.639 | 1.000 | 0.90 (0.58-1.39) |
|  | rs6457452 | TT+CT vs. CC | 7/143 | 22/161 | **0.018** | **0.036** | 0.36 (0.15-0.86) |
| *HSP70-hom* | rs1043618 | CC+CG vs. GG | 80/70 | 93/90 | 0.648 | 1.000 | 1.11 (0.72-1.70) |
|  | rs2227956 | GG+AG vs. AA | 60/90 | 74/109 | 0.935 | 0.935 | 0.98 (0.63-1.53) |
| *TNF-α* | rs361525 | AA+AG vs. GG | 3/147 | 4/179 | 1.000 | 1.000 | 0.91 (0.20-4.15) |
|  | rs1800629 | AA+GA vs. GG | 32/118 | 21/162 | **0.014** | 0.070 | 2.09 (1.15-3.81) |
|  | rs1799724 | TT+CT vs. CC | 27/123 | 40/143 | 0.382 | 0.955 | 0.79 (0.46-1.35) |
|  | rs1799964 | CC+CT vs. TT | 3/147 | 5/178 | 0.734 | 0.918 | 0.73 (0.17-3.09) |
|  | rs1800630 | AA+CA vs. CC | 40/110 | 44/139 | 0.583 | 0.972 | 1.15 (0.70-1.89) |
| *TNF-β* | rs909253 | AA+GA vs. GG | 92/58 | 134/49 | **0.021** | 0.053 | 0.58 (0.37-0.92) |
|  | rs1041981 | CC+AC vs. AA | 92/58 | 134/49 | **0.021** | **0.035** | 0.58 (0.37-0.92) |
|  | rs2857709 | AA+GA vs. GG | 1/149 | 5/178 | 0.228 | 0.285 | 0.24 (0.03-2.07) |
|  | rs2844484 | AA+GA vs. GG | 63/87 | 102/81 | **0.013** | 0.065 | 0.58 (0.37-0.89) |
|  | rs2229092 | CC+AC vs. AA | 2/148 | 6/177 | 0.303 | 0.303 | 0.40 (0.08-2.01) |
| *CFB* | rs641153 | AA+GA vs. GG | 13/137 | 21/162 | 0.400 | 0.800 | 0.73 (0.35-1.52) |
|  | rs4151667 | AA+TA vs. TT | 4/146 | 6/177 | 1.000 | 1.000 | 0.81 (0.22-2.92) |
| *C2* | rs9332739 | CC+CG vs. GG | 4/146 | 6/177 | 1.000 | 1.000 | 0.81 (0.22-2.92) |
|  | rs547154 | TT+GT vs. GG | 13/137 | 21/162 | 0.400 | 0.800 | 0.73 (0.35-1.52) |

*P* value was calculated using Chi-squared test or Fisher’s exact test. PSS: Posner-Schlossman syndrome; *P*: *P* value; *P_c_*: corrected *P* value; *CI*: confidence interval; *OR*: odds ratio; *P* values less than 0.05 are bolded.
