# Supplementary material for: HSP70 and TNF Loci Polymorphism Associated with the Posner-Schlossman Syndrome in a Southern Chinese Population
Source: J Immunol Res. 2022 Dec 9;2022:5242948. doi: 10.1155/2022/5242948 (PMC9757935; doi:10.1155/2022/5242948)
Supplement: Supplementary Materials — Supplementary Table 1: characteristic information, product size, and primers of the SNPs in HLA-III genes. Supplementary Table 2: other HLA-III allele frequencies in PSS cases and controls. Supplementary Table 3: other HLA-III haplotype frequencies between PSS patients and healthy controls. Supplementary Table 4: dominant genetic models of HLA-III gene in PSS cases and controls. Supplementary Table 5: recessive genetic models of HLA-III gene in PSS cases and controls. Supplementary Table 6: additive genetic models of HLA-III gene in PSS cases and controls. (Supplementary Materials) [file 5242948.f1.zip › Supplementary Table 5 (1).docx]

**Supplementary Table 5. Recessive genetic models of *HLA-Ⅲ* gene in PSS cases and controls**

| Gene | Variants | Recessive | PSS | Control | *P* | *P_c_* | *OR (95%CI)* |
| --- | --- | --- | --- | --- | --- | --- | --- |
| *HSP70-1* | rs1008438 | AA+CA vs. CC | 127/23 | 153/30 | 0.792 | 0.792 | 1.08 (0.60-1.96) |
|  | rs562047 | GG+CG vs. CC | 149/1 | 183/0 | 0.450 | 0.900 | 0.45 (0.40-0.51) |
|  | rs12190359 | CC+CT vs. TT | 150/0 | 183/0 | / | / | / |
| *HSP70-2* | rs2763979 | CC+CT vs. TT | 143/7 | 176/7 | 0.703 | 0.703 | 0.81 (0.28-2.37) |
|  | rs6457452 | CC+CT vs. TT | 149/1 | 183/0 | 0.450 | 0.900 | 0.45 (0.40-0.51) |
| *HSP70-hom* | rs1043618 | GG+CG vs. CC | 133/17 | 160/23 | 0.730 | 0.730 | 1.13 (0.58-2.19) |
|  | rs2227956 | AA+AG vs. GG | 142/8 | 169/14 | 0.397 | 0.794 | 1.47 (0.60-3.61) |
| *TNF-α* | rs361525 | GG+AG vs. AA | 150/0 | 183/0 | / | / | / |
|  | rs1800629 | GG+GA vs. AA | 149/1 | 182/1 | 1.000 | 1.000 | 0.82 (0.05-13.20) |
|  | rs1799724 | CC+CT vs. TT | 148/2 | 178/5 | 0.464 | 1.000 | 2.08 (0.40-10.87) |
|  | rs1799964 | TT+CT vs. CC | 147/3 | 178/5 | 0.734 | 0.979 | 1.38 (0.32-5.86) |
|  | rs1800630 | CC+CA vs. AA | 148/2 | 178/5 | 0.464 | 0.928 | 2.08 (0.40-10.87) |
| *TNF-β* | rs909253 | GG+GA vs. AA | 128/22 | 145/38 | 0.150 | 0.225 | 1.53 (0.86-2.71) |
|  | rs1041981 | AA+AC vs. CC | 128/22 | 145/38 | 0.150 | 0.150 | 1.53 (0.86-2.71) |
|  | rs2857709 | GG+GA vs. AA | 150/0 | 183/0 | / | / | / |
|  | rs2844484 | GG+GA vs. AA | 143/7 | 166/17 | 0.105 | 0.315 | 2.09 (0.84-5.19) |
|  | rs2229092 | AA+AC vs. CC | 150/0 | 183/0 | / | / | / |
| *CFB* | rs641153 | GG+GA vs. AA | 150/0 | 181/2 | 0.503 | 0.503 | / |
|  | rs4151667 | TT+TA vs. AA | 150/0 | 183/0 | / | / | / |
| *C2* | rs9332739 | GG+CG vs. CC | 150/0 | 183/0 | / | / | / |
|  | rs547154 | GG+GT vs. TT | 150/0 | 181/2 | 0.503 | 0.503 | / |

*P* value was calculated using Chi-squared test or Fisher’s exact test. PSS: Posner-Schlossman syndrome; *P*: *P* value; *P_c_*: corrected *P* value; *CI*: confidence interval; *OR*: odds ratio.
