# Supplementary material for: HSP70 and TNF Loci Polymorphism Associated with the Posner-Schlossman Syndrome in a Southern Chinese Population
Source: J Immunol Res. 2022 Dec 9;2022:5242948. doi: 10.1155/2022/5242948 (PMC9757935; doi:10.1155/2022/5242948)
Supplement: Supplementary Materials — Supplementary Table 1: characteristic information, product size, and primers of the SNPs in HLA-III genes. Supplementary Table 2: other HLA-III allele frequencies in PSS cases and controls. Supplementary Table 3: other HLA-III haplotype frequencies between PSS patients and healthy controls. Supplementary Table 4: dominant genetic models of HLA-III gene in PSS cases and controls. Supplementary Table 5: recessive genetic models of HLA-III gene in PSS cases and controls. Supplementary Table 6: additive genetic models of HLA-III gene in PSS cases and controls. (Supplementary Materials) [file 5242948.f1.zip › Supplementary Table 6 (1).docx]

**Supplementary Table 6. Additive genetic models of *HLA-Ⅲ* gene in PSS cases and controls**

| Gene | Variants | Additive | PSS | Control | *P* | *P_c_* | *OR (95%CI)* |
| --- | --- | --- | --- | --- | --- | --- | --- |
| *HSP70-1* | rs1008438 | AA vs. CC | 54/23 | 68/30 | 0.916 | 0.916 | 1.04 (0.54-1.98) |
|  | rs562047 | GG vs. CC | 120/1 | 146/0 | 0.453 | 0.906 | / |
|  | rs12190359 | CC vs. TT | 149/0 | 171/0 | / | / | / |
| *HSP70-2* | rs2763979 | CC vs. TT | 85/7 | 99/7 | 0.783 | 0.783 | 0.86 (0.29-2.55) |
|  | rs6457452 | CC vs. TT | 143/1 | 161/0 | 0.472 | 0.944 | 0.47 (0.42-0.53) |
| *HSP70-hom* | rs1043618 | GG vs. CC | 70/17 | 90/23 | 0.887 | 0.887 | 1.05 (0.52-2.12) |
|  | rs2227956 | AA vs. GG | 90/8 | 109/14 | 0.427 | 0.854 | 1.45 (0.58-3.60) |
| *TNF-α* | rs361525 | GG vs. AA | 147/0 | 179/0 | / | / | / |
|  | rs1800629 | GG vs. AA | 118/1 | 162/1 | 1.000 | 1.000 | 0.73 (0.05-11.76) |
|  | rs1799724 | CC vs. TT | 123/2 | 143/5 | 1.000 | 1.000 | 1.43 (0.34-6.12) |
|  | rs1799964 | TT vs. CC | 109/3 | 136/5 | 1.000 | 1.000 | 1.13 (0.31-5.71) |
|  | rs1800630 | CC vs. AA | 110/2 | 139/5 | 0.473 | 1.000 | 1.98 (0.38-10.39) |
| *TNF-β* | rs909253 | GG vs. AA | 58/22 | 49/38 | **0.029** | 0.087 | 2.05 (1.07-3.91) |
|  | rs1041981 | AA vs. CC | 58/22 | 49/38 | **0.029** | **0.044** | 2.05 (1.07-3.91) |
|  | rs2857709 | GG vs. AA | 149/0 | 178/0 | / | / | / |
|  | rs2844484 | GG vs. AA | 87/7 | 81/17 | **0.038** | **0.038** | 2.61 (1.03-6.62) |
|  | rs2229092 | AA vs. CC | 148/0 | 177/0 | / | / | / |
| *CFB* | rs641153 | GG vs. AA | 137/0 | 162/2 | 0.502 | 0.502 | / |
|  | rs4151667 | TT vs. AA | 146/0 | 177/0 | / | / | / |
| *C2* | rs9332739 | GG vs. CC | 146/0 | 177/0 | / | / | / |
|  | rs547154 | GG vs. TT | 137/0 | 162/2 | 0.502 | 0.502 | / |

*P* value was calculated using Chi-squared test or Fisher’s exact test. PSS: Posner-Schlossman syndrome; *P*: *P* value; *P_c_*: corrected *P* value; *CI*: confidence interval; *OR*: odds ratio; *P* values less than 0.05 are bolded.
